# Integration of IoT Technologies into the Smart Grid

**DOI:** 10.3390/s22072475

**Published:** 2022-03-23

**Authors:** Salvatore Cavalieri, Giulio Cantali, Andrea Susinna

**Affiliations:** 1 Department of Electrical Electronic and Computer Engineering, University of Catania, 95125 Catania, Italy; 2 COMETA Consortium, S. Sofia 64, 95123 Catania, Italy; giulio.cantali@consorzio-cometa.it (G.C.); andrea.susinna@consorzio-cometa.it (A.S.)

**Keywords:** IEC 61850, Web Services, REST, MQTT, Internet of Things, Smart Grid

## Abstract

This paper presents a novel solution in the field of the integration of the Smart Grid and the Internet of Things. The definition of a web platform able to offer a RESTful interface to IEC 61850 Servers to a generic user is proposed. The web platform enables the mapping of information maintained by an IEC 61850 Server into MQTT messages. Suitable mechanisms to introduce interoperable exchange of information were defined. The paper presents the main features offered by the proposed platform. The originality of the proposal is highlighted by comparing it with the current literature. A prototype was realized, and the software implementation choices are described and the main results of its evaluation are presented.

## 1. Introduction

The Smart Grid (SG) is based on a new vision of the electric grid, which includes the maximization of the distribution of energy demand, the minimization of losses and the integration of renewable energy sources on a large scale, as pointed out in [1,2,3]. The SG aims to overcome one of the main limitations of the current electric grid, related to the lack of integration of novel information and communication technologies.

The current information and communication technologies include those based on the Internet of Things (IoT), which plays a strategic role in the era of Industry 4.0, as shown by [4,5,6,7,8]. The eXtensible Messaging Presence Protocol (XMPP) and the Message Queue Telemetry Transport (MQTT) are some of the most well-known protocols in this field [9,10,11]. The REpresentational State Transfer (REST) is an architectural style defined by Roy Fielding in his PhD dissertation [12]; a web service based on REST is called a RESTful Web Service [13]. The RESTful Web Service is a paradigm that can enhance the IoT application scope by making smart things part of the web [14].

The paper proposes a novel solution in the field of the integration of SG and IoT. In particular, the proposed integration involves IEC 61850, which is an international standard defining communication protocols for intelligent electronic devices at electrical substations [15,16]. The definition of a web platform able to offer to a generic web user a RESTful interface to IEC 61850 Servers is proposed. The web platform is based on a REST architecture and enables the mapping of IEC 61850 information model into MQTT messages. The platform is called the *IEC 61850 Web Platform* in the remainder of the paper.

The paper is organized as follows. The next section provides an overview of the current state-of-the-art research, and highlights the originality of the proposal. Section 3 introduces the main features of the IEC 61850, focusing on the relevant data model. Section 4 describes the IEC 61850 Web Platform in detail. Section 5 presents the implementation details of the prototype realized by the authors. The final section summarizes the main results.

## 2. Related Work

The current literature presents several studies about enabling IoT in the SG, typically referred as IoT-enabled SG [17,18,19,20].

In [21], the authors present a review of the IoT protocols used in the SG aiming to achieve guidelines in utilizing a proper IoT protocol that can meet the SG requirements; addressing effective elements in applying IoT in the SG’s future trends is another contribution of this paper. In [22], the authors discuss integration of IEC 61850 and IoT. In [23], an IEC 61850 and XMPP communication-based energy management in microgrids with integrated electric vehicles is introduced. In [24], the XMPP and MQTT are applied to an IEC 61850-based grid, and the most suitable protocol for the microgrid environment is derived by comparing two protocols.

Among the main approaches aimed at enabling interworking between REST and SG domains, in [25] a mapping between CoAP and IEC 61850-based substation automation systems in an SG environment is proposed. Based on REST, CoAP is a specialized web transmission protocol for use with constrained nodes and constrained networks. Another solution for the mapping of the IEC 61850 to RESTful Web Services is presented in [26].

Based on the state-of-the art research presented above, it can be noted that the current literature features some approaches aiming to integrate the IEC 61850 standard with IoT technologies, such as RESTful Web Services and MQTT. The motivation of this paper was to contribute to the state-of-the-art research in the definition of a solution able to integrate an IEC 61850-based Smart Grid and IoT, thus overcoming the limitations of the current solutions. The main feature of the proposed solution is the co-existence of the following mechanisms.

First, a RESTful interface to IEC 61850 Servers is available, allowing a generic user to access the information maintained by the IEC 61850 Server by a REST data exchange. Then, a mechanism to map each information exchanged with the IEC 61850 Server into JSON format was defined. Distribution of this information through a publisher/subscribe communication model based on the use of an MQTT Broker was also defined.

Finally, a mechanism to enable the interoperability of the data exchange between a generic user and the IEC 61850 Server is included in the proposed solution. To allow the user to encode/decode each piece of information exchanged with the IEC 61850 Server through the IEC 61850 Web Platform, particular services were defined to allow the user to acquire knowledge about the data type descriptions of the data maintained by the IEC 61850 Server. The services here defined allow the user to retrieve, in the JSON standard format, the description of data types relevant to the information maintained in the IEC 61850 Server.

To the best of the authors’ knowledge, the current literature does not present a solution that includes all of the entire set of features explained above. The authors believe that the combination of these features is clearly needed if a real integration between the Smart Grid and IoT is to be achieved. In the following discussion, the reasons for this requirement are clearly highlighted.

The RESTful interface was chosen in this research mainly due to the results available in the current literature, which indicate the superiority of the RESTful interface compared to other available solutions. The RESTful interface is broadly used, as the functionality of RESTful services is very closely related to the functionality of the web; this is mainly due to its scalability, interoperability, and the fact that it is simple and easy to understand [26]. RESTful APIs are widely being used in the modern web, and it is a good model for heterogeneous systems [27]. Web services are categorized into two categories: REST and Simple Object Access Protocol (SOAP). The performance of REST is superior to that of SOAP [28]. RESTful APIs are directly connected with IoT devices as they allow the safe and authorized exposure of devices to clients, and various appliances in the IT framework. To fully benefit from the IoT, a RESTful API is required for each IoT device, because REST ensures the smooth data transfer over network regulations and controls the authorized and secured transactions. A RESTful API portrays a large number of classes of IoT applications very well [28].

The adoption of a publisher/subscribe communication model based on the use of an MQTT Broker for the distribution of information maintained by an IEC 61850 Server is also a very important feature. IEC 61850 communication is based on a request/response model; the IEC 61850 Client must issue a sequence of requests to the Server in order to receive the updates of a particular piece of information. The use of an MQTT Broker allows the user to receive updates each time a value changes, or each time a certain time interval elapses. This improves the efficiency of the exchange of information with a server.

Mapping each piece of information exchanged with the IEC 61850 Server into the JSON format is also very important; this allows a generic user to receive information in an open format instead of receiving it according to the IEC 61850 protocol.

Finally, the mechanism to allow the user to acquire knowledge about the data type descriptions of the data maintained by the IEC 61850 Servers seems to be strategic because interoperability is a very important feature in the IoT. It is important to point out that this feature is totally missing in all the other approaches available in the current literature.

## 3. IEC 61850 Data Model

IEC 61850 is the leading standard for substation automation [15,16]. It is intended to structure the intelligence of protection, control, monitoring, and automation functions. The standard fulfils automation architecture requirements for utility subsystems, enabling communication among multi-vendor equipment.

IEC 61850 defines an object-oriented modeling of process data required for power system automation. The IEC 61850 data model has been primarily defined for the exchange of information within substations by the document IEC 61850-7-4 [15]. The IEC 61850 data model has been extended for other domains, including distributed energy resources (DERs) by IEC 61850-7-420 [15].

**Figure 1:**
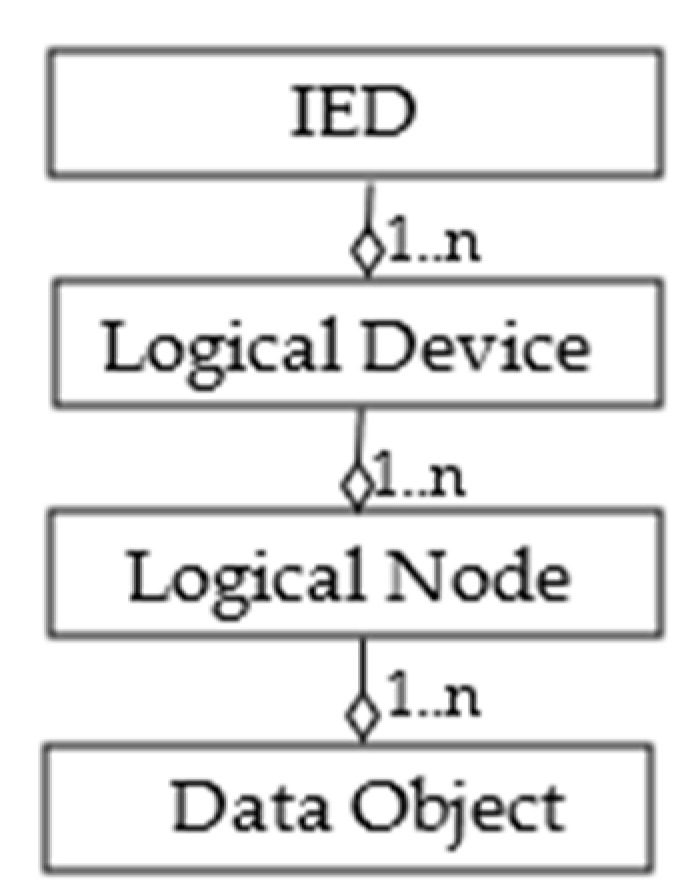
IEC 61850 Data Model.

Figure 1 shows the main elements of the IEC 61850 data model. The top parent class is the intelligent electronic device (IED), which is hosted by physical device, i.e., the controller part of the device.

**Figure 2:**
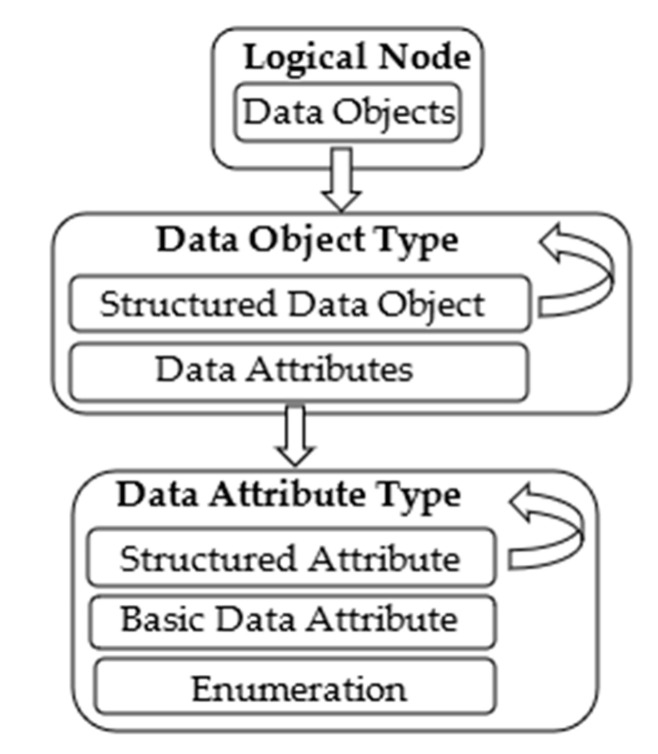
IEC 61850 data type template hierarchy.

The IED consists of one or more Logical Devices (LDs), i.e., virtual representations of devices intended for supervision, protection, or control of automated systems. LDs are created by combining several Logical Nodes (LNs), which represent various device functionality interfaces. Data Objects (DOs) are representations of the common information of Logical Nodes. DOs are categorized to common data classes (CDCs) from IEC 61850-7-3 or IEC 61850-7-2 [15]. Each CDC describes the type and structure of the data object within the LN. A Data Object belongs to the Data Object Type; Figure 2 gives the details of the hierarchical structure of the Data Object Type. It may contain Data Attributes and a Structured Data Object (SDO), belonging to the Data Attribute Type and to the Structured Data Object Type, respectively. Like the Data Object, Structured Data Objects are also defined by Data Object Types, as shown by Figure 2.

A Data Attribute is defined by a Data Attribute Type. It may be a Structured Attribute, or it may belong to a customized Enumerator type or to a Basic Data Attribute (BDA) type. In the simplest case, a Basic Data Attribute may be of a basic type, such as Integer, Floating Point, or Boolean. Like the Data Attribute, a Structured Attribute is of the Data Attribute Type.

According to the IEC 61850-7-1 [15], the IED is identified by the attribute *name*; a Logical Device inside the IED is identified by the attribute *inst*. Each Logical Node is identified by a concatenation of three attributes: *prefix*, *lnClass* (Logical Node Class), and *inst* (Logical Node Instance); in particular, this concatenation is made up by the sequence of the values of the three attributes in the same order as shown before. Finally, Data Objects, Structured Data Objects, Structured Attributes, Basic Data Attributes, and Enumeration are univocally identified by the attribute *name*.

**Figure 3:**
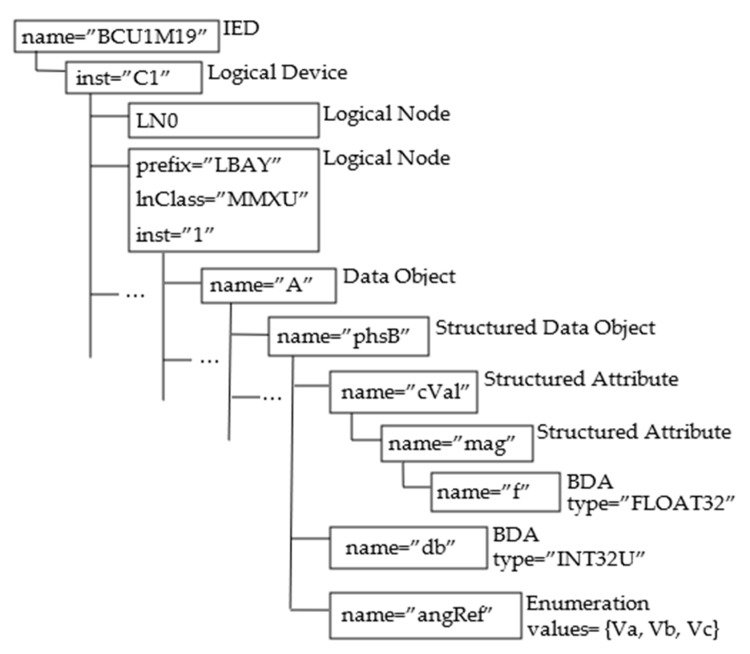
Example of the IEC 61850 data model used throughout the paper.

In order to better understand the IEC 61850 hierarchical data model, an example is given, and is used throughout the paper. Figure 3 shows the example of the IEC 61850 data model considered here. It is made up of an IED whose attribute name has the value “BCU1M19”; the IED contains a Logical Device whose attribute inst is “C1”. Among the Logical Nodes belonging to this Logical Device, let us assume the existence of the LN featured by: prefix = “LBAY”, lnClass = “MMXU”, and inst = ”1”; according to this hypothesis, the LN is identified by the concatenation “LBAYMMXU1”. Among the Data Objects inside this LN, let us assume the presence of the Data Object with the attribute name set to “A”, containing a Structured Data Object with the name = “phsB”. It has been assumed that it includes a Basic Data Attribute (BDA) with the name = “db”, represented by an unsigned 32-bit integer (INT32U) type. Then, an enumeration is present with the name = “angRef”; Figure 3 shows the enum values. The Structured Data Object with the name = “PhsB” also contains a Structured Attribute featuring the attribute name set to “cVal”; it is made up of another Structured Attribute with the attribute name equal to “mag”. This attribute contains only a Basic Data Attribute with the name = “f”, represented by a 32-bit real number (FLOAT32) type.

The IEC 61850 standard provides the representation of an IEC 61850 system configuration of electrical devices, including representation of data and communication services, using an XML-based file format called Substation Configuration Language (SCL). The SCL file is divided into sections, each of which provides different information. The header section includes general topics and details about SCL and the file version. The IED section describes devices capacities, functions, and data it manages, and provides a description of the Logical Devices, Logical Nodes, and Data Objects present in the system. The DataTypeTemplates section describes the data model by defining the Logical Node Types, the Data Object Types, the Data Attribute Types, and the Enumeration Types used. The communication section contains communication details. The substation section describes electrical topology for substation or any other electrical process.

The IED Capability Description (ICD) file is a specific type of Substation Configuration Language (SCL) file, containing a generic description of the whole capability range of a given device, including the functions and objects it can support. The ICD file is usually supplied by the developer/manufacturer.

IEC 61850 standardizes the set of abstract communication services that allows the compatible exchange of information among components of a Power Utility Automation System. IEC 61850 offers several types of communication models, including the Client/Server type communication services model [15]. An IEC 61850 Server may be used to maintain, and make its content available to, IEC 61850 Clients, in terms of data structured according to the IEC 61850 data model shown by Figure 1 and Figure 2. An ICD file is always available in the IEC 61850 Server to describe its full content.

## 4. IEC61850 Web Platform

**Figure 4:**
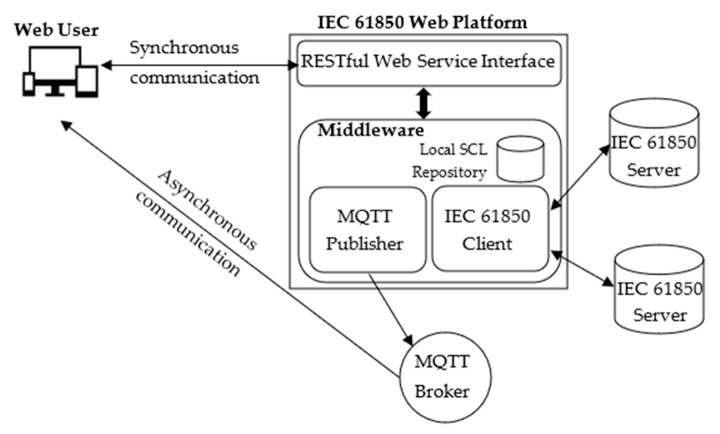
Architecture of the proposed IEC 61850 Web Platform.

The IEC 61850 Web Platform proposed here is based on the adoption of a REST architectural style and is shown in Figure 4. As can be seen, it offers access to one or more IEC 61850 Servers to a Web User.

The term Web User is used in this paper to refer to a generic application running on a generic device which consumes the services offered by the IEC 61850 Web Platform. It is constrained neither to be IEC 61850 compliant nor to implement the IEC 61850 communication stack. The only constraints are the adoption of RESTful-based communication and the MQTT-based exchange of information.

The RESTful Web Service Interface shown by Figure 4 accepts requests submitted by a registered and authenticated Web User.

The Middleware module inside the IEC 61850 Web Platform performs all the operations needed to fulfil each request coming from a Web User. These requests may require data exchange with the available IEC 61850 Servers; for this reason, the Middleware includes an IEC 61850 Client used for the access to the IEC 61850 Servers. Communication between an IEC 61850 Client and IEC 61850 Servers occurs according to the standard IEC 61850 communication protocol and services (i.e., using the IEC 61850 communication stack) [15]. The Middleware includes a particular module named MQTT Publisher, which is in charge of publishing information taken from the IEC 61850 Server, through an MQTT Broker, using the MQTT protocol. The Middleware is made up of a local repository shown by Figure 4 (i.e., Local SCL Repository), used to store ICD files containing the SCL description of the data model maintained by each IEC 61850 Server involved in the data exchanges with Web Users. This description is needed by the IEC 61850 Web Platform to accomplish the services offered, as explained in the remainder of this section. The use of this local repository is not mandatory, but it is very useful because it avoids remote queries to the IEC 61850 Server when information about the SCL description of the relevant data model is needed.

Communication between a Web User and the IEC 61850 Web Platform may be synchronous (based on RESTful web services) or asynchronous (based on MQTT Publish/Subscribe Pattern), as shown in Figure 4.

The Web User uses synchronous communication to interact with the Web Service Interface to request one of the services offered by the IEC 61850 Web Platform. POST requests are realized using the JSON format. For each Web User’s request through the RESTful Web Service Interface, the IEC 61850 Web Platform will send a relevant response. Each Web User needing to use the IEC 61850 Web Platform through synchronous communication must be previously registered (by user credentials in terms of username and password) and authenticated (through the use of a signed token which is held by the Web User).

Asynchronous communication is realized through the use of MQTT Brokers to allow the Web User to receive data produced by the platform; data is mainly originated by the IEC 61850 Servers. The Web User must be registered to a particular topic in order to receive the information needed from the relevant MQTT Broker. The Broker may be chosen by the Web User, or he can use a given predefined Broker.

The following subsections provide more details about the IEC 61850 Web Platform. In particular, all the operations performed by the Middleware and its internal components are pointed out; the authors preferred to detail the functionalities of the Middleware for each single service offered by the RESTful Web Service Interface and requested by the Web User. Before this description, Section 4.1 provides an overview of a particular syntax defined to allow the Web User to univocally identify each element of the IEC 61850 data model.

### 4.1. Definition of a Syntax for the Web User

In Section 3, it was noted that each piece of information in the IEC 61850 Server is maintained according to the hierarchical data model structure shown by Figure 1 and Figure 2; in particular, Figure 2 gives the details about the data type structure.

To allow the Web User to access the elements of this data model, a syntax had to be defined to univocally identify each IEC 61850 element; it was assumed to adopt the IEC 61850 conventions as much as possible. For each element present in the IEC 61850 data model, the relevant identifier from the Web User point of view is defined as a sequence of names separated by “/”. The sequence of names is defined according to the same hierarchical order shown by Figure 1 and Figure 2. Thus, the identifier must first foresee the attribute name of the IED. Then, the value of the attribute inst of the Logical Device contained in the IED is used after the IED attribute name. In Section 3, it was noted that the IEC 61850 standard identifies each Logical Node by a particular concatenation of three attributes: prefix, lnClass, inst; for this reason, it was assumed that this concatenation is used after the LD identifier. As Data Objects, Structured Data Objects, Basic Data Attributes, and Enumeration are univocally identified by the attribute name according to the IEC 61850 standard, the value of this attribute is used in the identifier according to the hierarchical order shown by Figure 1 and Figure 2.

On the basis of the syntax just described and considering the example of the IEC 61850 data model depicted by Figure 3, the identifier of the BDA with the name = “f” becomes “BCU1M19/C1/LBAYMMXU1/A/phsB/cVal/mag/f”; the identifier of the BDA with the name = “db” is “BCU1M19/C1/LBAYMMXU1/A/phsB/db”.

### 4.2. Web User Registration and Authentication

Use of the IEC 61850 Web Platform by a Web User may occur only after his registration. If the user is not yet registered in the system, he must issue a suitable POST request to a specific Web Service Interface, the body of which must contain, in JSON format, the credentials (username and password) with which the user intends to register. If the registration procedure succeeds, the Web User will receive a POST response, again in JSON, indicating that the operation was completed successfully. If registration procedure fails (e.g., if the user is already present in the system), the POST response will specify the failure reason.

Before the registered Web User may proceed to request synchronous services, he must authenticate himself to the platform. The user will send a POST request to another specific Web Service Interface relevant to the authentication service, specifying his personal credentials used during the registration. If the authentication request succeeds, the Web User will receive a response containing a signed token to be used in each of the next Web Service synchronous requests issued to the IEC 61850 Web Platform.

### 4.3. Access to an IEC61850 Server

The main aim of the IEC 61850 Web Platform is to allow the access to the IEC 61850 Server from the web. Access means that the Web User may request to retrieve and/or to update information maintained by a particular IEC 61850 Server. To achieve this goal, the Web User must pass to the platform the information about the IEC 61850 Server he desires to access; this must be realized only one time, after registration and authentication, before the data exchanges between Web User and the server can occur. A POST request must be issued at a particular Web Service Interface, mainly including the IP address of the remote machine hosting the IEC 61850 Server and the port on which the server is listening.

In Section 2, it was noted that each IEC 61850 Server holds a particular file (i.e., the ICD file) containing the SCL description of the data model maintained by the server. This description is needed by the IEC 61850 Web Platform to accomplish some services, as explained in the following subsections. For this reason, on receipt of the Web User’s request, the Middleware connects to the server specified by the Web User in order to retrieve the relevant ICD file and store it in the local SCL repository shown by Figure 4. This operation is not performed if the Middleware realizes that the same server is used by other Web Users and the relevant ICD file has been already retrieved and stored in the local SCL repository; in this case the same ICD file is associated to more Web Users. On the completion of this operation, the IEC 61850 Web Platform will respond with a POST response, pointing out the success of the previous Web User request.

A disconnection procedure has been foreseen through a specific POST request issued by the Web User, when the data exchanges with a specific IEC 61850 Server end. All the local resources allocated to maintain the SCL file descriptions of the IEC 61850 Server data model accessed by the Web User are released if not used by other users.

### 4.4. Publication of Data Types

The main requirements in an interoperable data exchange include the capability to properly decode each piece of information received from the counterpart. To enable each value received to be properly decoded by the receiving entity, the relevant data type must be known. Knowledge of the data type to which each variable exchanged belongs is of paramount importance. The Web User must be able to encode/decode each piece of information exchanged with the IEC 61850 Server through the IEC 61850 Web Platform, and this can be achieved only on the basis of the knowledge of the data types involved in the information exchange. This means that the data type descriptions of the data maintained in the IEC 61850 Server must be known to the Web User, or at least the subset of data of his interest.

For this reason, a particular service was defined that allows a Web User to retrieve the description of the entire set (or a subset) of data types maintained by each IEC 61850 Server that is relevant to the information the Web User needs to retrieve from the IEC 61850 Server. A suitable POST request can be issued to a specific Web Service Interface, through which the Web User will specify the data types requested and the Broker to be used for the publication of these data types. The requested data type is described using the syntax introduced in Section 4.1. For each POST request received, the IEC 61850 Web Platform will explore the Local SCL Repository in order to find the information related to the IEC 61850 data types requested by the user. A JSON frame is built containing the description of these data types; this frame will be published through MQTT message by the requested Broker, on a particular topic which is passed to the Web User through the POST response. Subscription to this topic by the Web User allows him to receive the requested data type description though a JSON payload inside the MQTT frame received.

**Figure 5:**
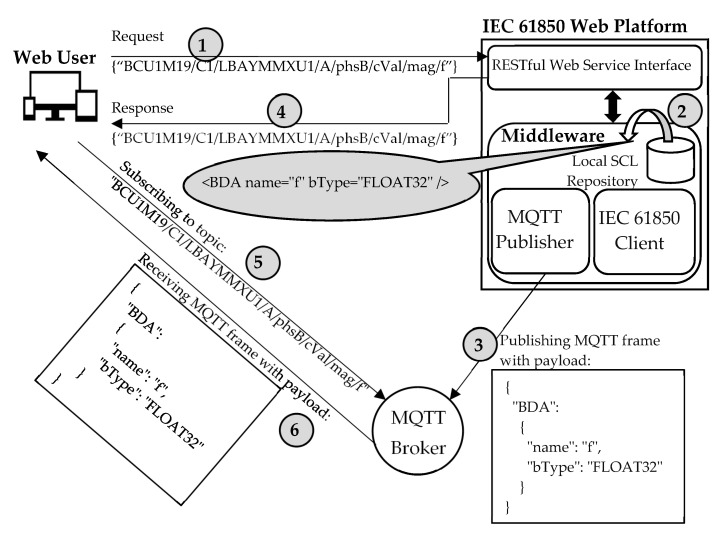
Example of the publication of the description of a Basic Data Attribute Type.

Figure 5 shows an example of the above description; in particular, Figure 5 shows the steps that enable the Web User to retrieve the description of the data type of the BDA with the attribute name = f” shown by Figure 3. According to the syntax described in Section 4.1, this BDA is identified by the string “BCU1M19/C1/LBAYMMXU1/A/phsB/cVal/mag/f”. These steps are represented inside numbered circles, as shown in Figure 5; the numbers are assigned according to the temporal sequence of the steps. In the first step, the Web User submits a POST request including the identifier of the BDA whose data type he is interested in and the details about the Broker to be used for the publication (this last piece of information is not shown in Figure 5 due to the lack of space). In order to accomplish the user’s request, the Middleware will analyze the SCL data model description maintained by the Local SCL Repository (step 2), looking for the SCL description of the BDA in the DataTypeTemplates section of the SCL file associated with the Web User. Figure 5 shows the piece of the SCL DataTypeTemplates section relevant to the requested BDA, inside the callout. The SCL description of this data type is translated into JSON format, thus obtaining the payload shown in Figure 5 close to the step number 3. This payload is published to the Broken chosen by the Web User. The IEC 61850 Web Platform will send the topic identifier as a POST response to the Web User (step 4). In step 5, the Web User will subscribe to this topic on the specified Broker. Subscription will allow the Web User to receive an MQTT frame containing the requested BDA data type in JSON format (step 6).

According to the example shown in Figure 5, the Web User must be aware of the identifier “BCU1M19/C1/LBAYMMXU1/A/phsB/cVal/mag/f” of the BDA featured by the attribute name = “f”. This happens in two scenarios; the first is the simplest and occurs when the Web User holds a list of pre-configured identifiers, for each of which he needs to know the relevant data type. The second scenario is that the Web User has a complete knowledge of the current structure of the hierarchical data model of the IEC 61850 server; on the basis of this knowledge, he is able to specify whatever identifier he desires. Sometimes these two scenarios cannot be realized, and the Web User may have to explore at run-time the current IEC 61850 data model maintained by a specific server, until he can find a particular data type and the relevant identifier. For this reason, the implementation of the proposed platform includes particular solutions to allow the Web User to acquire information about the current IEC 61850 data model present in the IEC 61850 Server.

If the Web User has the need to discover the full or partial structure of the hierarchical data model starting from a specific element (e.g., the IED), he will perform several POST requests in order to explore the entire data model, starting from the chosen starting element of the data model, until the requested data types are found. In order to achieve this, a particular syntax was defined to be used in these POST requests. This syntax is based on the same rules described in Section 4.1 for the definition of the name of the data type identifier; moreover, the syntax contains particular operators aimed to make the exploration of the data model easier.

**Figure 6:**
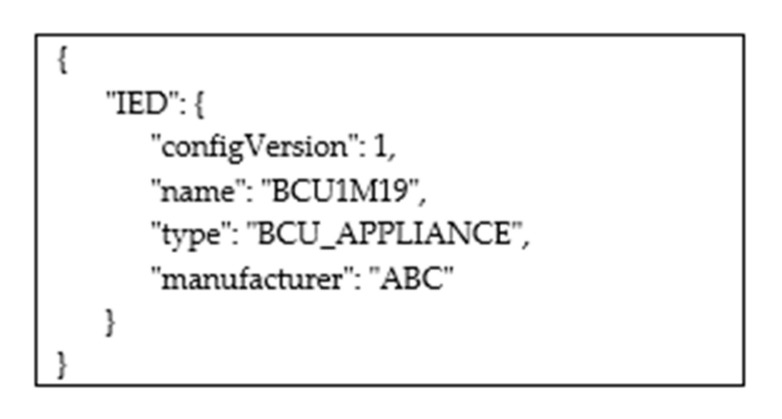
Example of a JSON frame containing the data type description of the IED maintained by the IEC 61850 Server.

**Figure 7:**
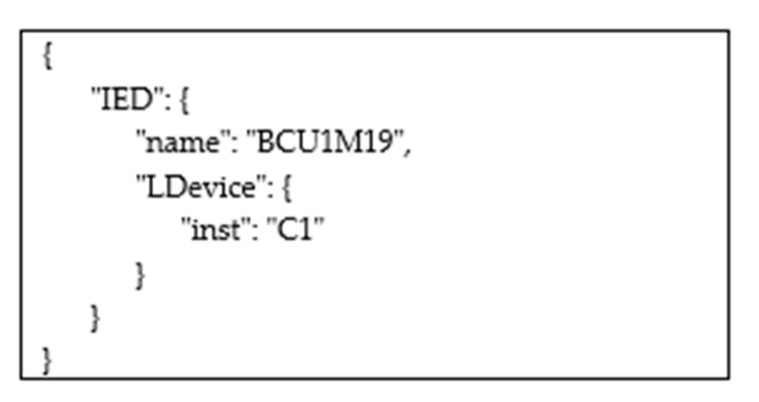
Example of the JSON frame containing the data type description of the LD belonging to the IED “BCU1M19”.

**Figure 8:**
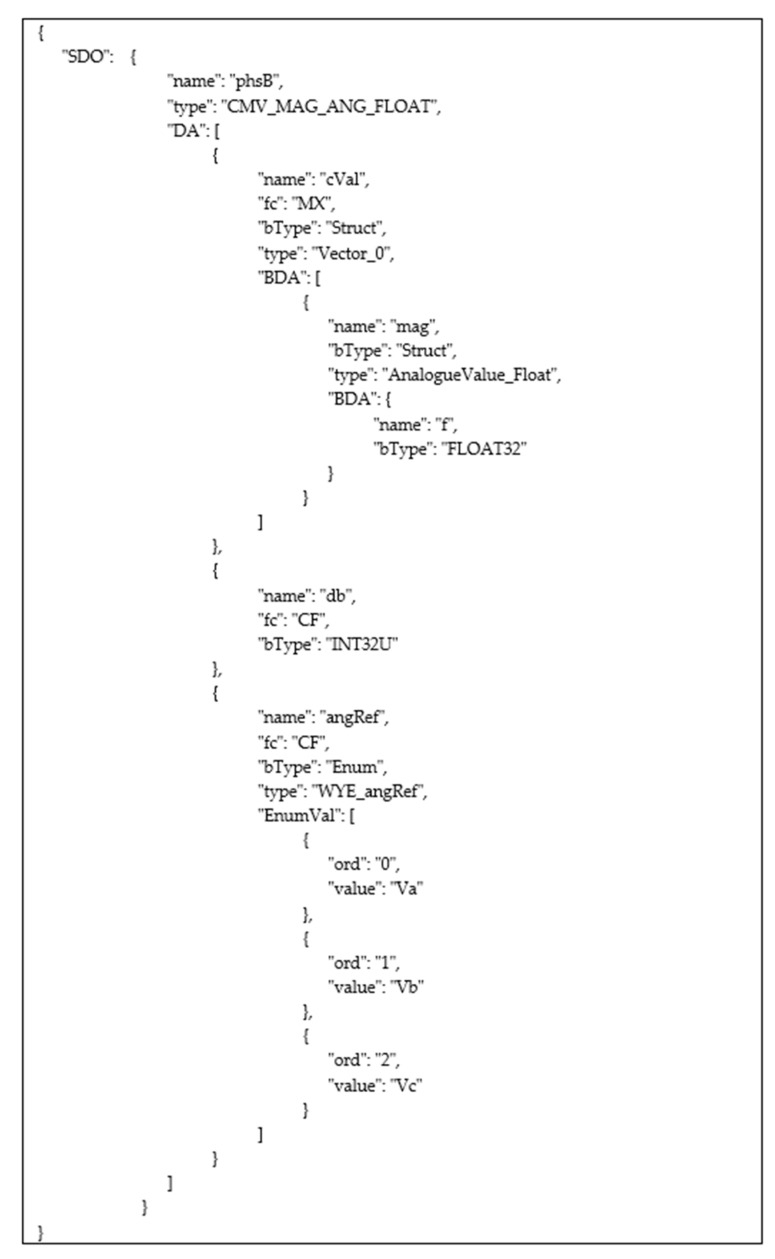
JSON frame containing the data type description of the SDO “phsB” and the relevant content.

The following operators were defined to enable the Web User to explore the hierarchical IEC 61850 data model. This is not an exhaustive list, as only the most important operators are described:

1. getAllIEDs: If the Web User specifies this operator in his request, it will receive a topic named “IEDs”, whose subscription to the proper Broker enables a JSON description of the IEDs maintained by the IEC 61850 Server. Considering the data model shown by Figure 3, the JSON frame shown by Figure 6 will be received through the subscription to this topic. Publication of this frame occurs as shown by Figure 5. In this way, the Web User discovers the value of the attribute name of the IED. This value will be used in all the following queries in order to explore the content of the data model below the IED elements.
2. getAllLogicalDevices: This operator allows the Web User to make a request for the publication of the data structure relevant to the entire set of LDs contained in a specific IED. Again considering the data model shown by Figure 3, the Web User may specify the string “BCU1M19/getAllLogicalDevices” in the POST request made to the platform. The topic he will receive from the Platform will be “BCU1M19/LDevices”; through the subscription to this topic. The JSON frame shown by Figure 7 will be received, with the description of the LDs contained in the IED. Again, the publication of this frame occurs according to the same sequence of steps shown by Figure 5. This allows the Web User to discover the value(s) of the attribute name(s) of the LDs included in an IED. This(these) value(s) will be used in all of the following queries to explore the data model below each LD.
3. getAllLogicalNodes: This allows the Web User to make a request for the publication of topics relevant to the entire set of LNs contained in a LD. Considering the example of Figure 3, the Web User may realize the following request “BCU1M19/C1/getAllLogicalNodes”. He will receive the topic “BCU1M19/C1/LNodes” from the Platform; through the subscription to this topic, the Web User will have the details about the LNs, including the LN whose identifier is “LBAYMMXU1”, as seen before, which can be used in the following queries.
4. getAllDataObjects: This allows the Web User to make a request for the publication of details relevant to the entire set of Data Objects contained in a Logical Node. Considering the example seen before, the Web User may perform a request specifying the string “BCU1M19/C1/LBAYMMXU1/getAllDataObjects”. The topic he will receive from the Platform will be “BCU1M19/C1/LBAYMMXU1/DObjects”; through the subscription to this topic, the Web User will receive the details about the DOs contained in the LN, including the DO with the attribute name = “A”.
5. getAllSDO: This is used in the request aimed at acquiring information about the entire set of Structured Data Objects and the relevant Data Attributes inside a Structured Data Object. Considering the example shown by Figure 3, let us assume that the Web User performs a POST request specifying the string “BCU1M19/C1/LBAYMMXU1/A/getAllSDO”. The Web User will receive the topic “BCU1M19/C1/LBAYMMXU1/A/SDO”, and through the relevant subscription, the Web User will receive the details (in JSON format) about the Structured Data Objects and the relevant content, as shown by Figure 8. As can be seen, the JSON content reflects the data model structure shown by Figure 3. As previously noted, the JSON frame is received by the Web User according to a sequence of steps very similar to those shown by Figure 5.
6. &: Through this operator, the Web User may request the publication of several elements. For example, he may specify the string “ied1/Inverter/getAllLogicalNodes & ied1/Battery/getAllLogicalNodes” in the POST request; in this way, the Web User requires knowledge of the structure of the Logical Nodes contained in the Inverter and Battery logical devices of the IED “ied1”.

### 4.5. Publications of Values

The Web User may require the current value of a particular data attribute maintained by an IEC 61850 Server. To enable each value received to be properly decoded by the receiving entity, the relevant data attribute type must be known; the previous section provides a detailed description of the services defined to allow the Web User to acquire information about the data type.

The Web User may need to request only the current value of a particular data attribute, or he may require receipt of updates of the value over time. For this reason, a particular service was defined to allow the Web User to retrieve the current value of a particular data attribute; moreover, the service may allow the Web User to request the automatic sampling of the data attribute and the transmissions of the sampled values with the same sampling frequency.

To use this service, the Web User sends a POST request to a particular Web Service Interface of the IEC 61850 Web Platform, containing three fields: identifier, interval, and broker.

Identifier contains the details about the data attribute whose value the Web User is interested in. It was assumed that the identifier is given according to the same formalism described in Section 4.1. For example, considering the example shown by Figure 3, the identifier “BCU1M19/C1/LBAYMMXU1/A/phsB/cVal/mag/f”, refers to the data attribute belonging to the Basic Data Attribute with the name = “f”. The operator & may be used in the POST request to specify a list of variables.

The interval field is used by the Web User to specify the desired sampling interval of the specified data attribute, in milliseconds; the sampling interval corresponds to the interval with which the IEC 61850 Client reads the requested data attribute from the server, and the relevant value is given back to the Web User through MQTT frames. If the Web User requires only one value (i.e., the current one), the interval must be set to “0”.

The information about the Broker is passed by the Web User if he needs to receive the requested value(s) on a specific Broker; otherwise, a default Broker is used by the IEC 61850 Web Platform.

**Figure 9:**
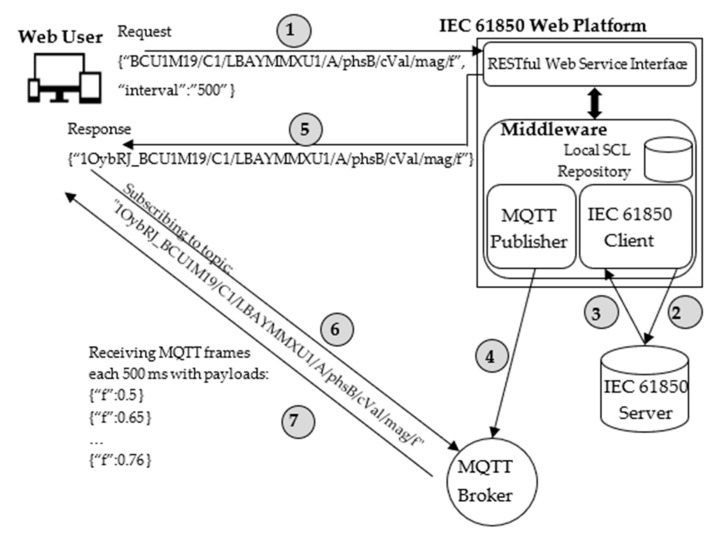
Example of periodic monitoring of a Basic Data Attribute.

Figure 9 shows an example of the service explained above. It is assumed that the Web User needs to receive the values of the BDA “BCU1M19/C1/LBAYMMXU1/A/phsB/cVal/mag/f”, sampled every 500 ms.

Figure 9 shows that the first step to be performed is the transmission of a POST request to a specific Web Service Interface, containing (in JSON format) the identifier of the data attribute and the sampling interval desired (the information about the Broker is not present due to the lack of space). According to this request, the IEC 61850 Client will proceed to read the requested value from the IEC 61850 Server using the IEC 61850 standard communication services. Figure 9 shows steps 2 and 3, relevant to the request of the current value from the server and the transmission of the requested value to the client, respectively. These steps are repeated over time on the basis of the interval specified by the Web User (only the current single value is retrieved if the interval value is “0”, as noted previously). For each value retrieved from the server, the Middleware creates a JSON file containing the value and the MQTT Publisher will send an MQTT frame containing this JSON file as the payload (step 4). The next step (step 5) is the transmission of the topic to be used by the Web User to retrieve the requested value(s) from the Broker. The Web User will subscribe to the Broker (step 6), and through this subscription the Web User will receive from the Broker the single value or the values updated according to the frequency specified (step 7); Figure 9 refers to this last scenario, noting the values sampled each 500 ms.

### 4.6. Updating Values in the IEC 61850 Server

The Web User may need to update the value of a data attribute in an IEC 61850 Server. A service was defined to achieve this goal. A POST request must be issued at a particular Web Service Interface, including the identifier of the data attribute to update and the relevant value. The Web User will receive a POST response to confirm the updating operation.

**Figure 10:**
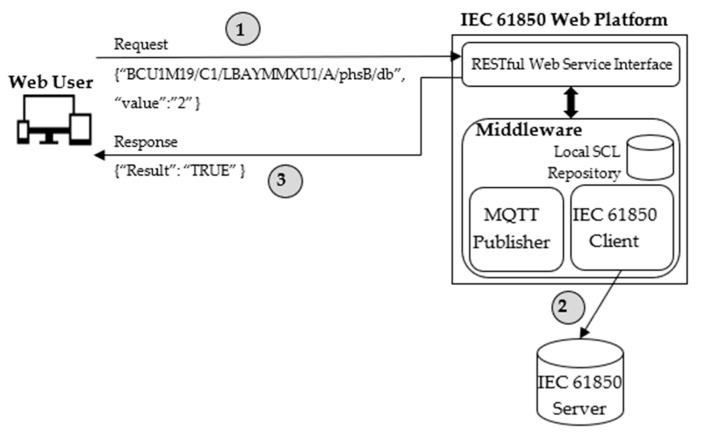
Example of updating of a value belonging to a Basic Data Attribute.

Figure 10 shows the several steps needed to accomplish the Web User’s request to update the value of the BDA identified by “BCU1M19/C1/LBAYMMXU1/A/phsB/db” (shown in Figure 3).

At the first step, a POST request is issued by the Web User containing the identifier of the data attribute and the relevant value to update. On the receipt of this request, the Middleware will perform the update through the IEC 61850 Client module; IEC 61850 communication services are used to update the specified data attribute with the value passed by the user (step 2). Once this operation has been successfully concluded, a POST response is returned to the Web User confirming the success of his previous request.

## 5. Software Implementation

The proposal presented here was developed inside a research project named PASCAL; details of the project are given in the Funding section.

The research project featured the implementation of a prototype of the IEC 61850 Web Platform according to the architecture depicted by Figure 4; furthermore, the prototype was tested in order to demonstrate the feasibility of the proposal. The aim of this section is to provide an overview of the software implementation choices and the details of the tests carried out.

The software implementation of the IEC 61850 Web Platform was based on the Java language.

For the Web User authentication, the standard JSON Web Token was used [29].

The Spring Boot framework [30] was used for the creation of the Web Service Interface shown by Figure 4.

The IEC 61860 Client inside the Middleware was implemented using the open-source library iec61850bean [31].

The conversions from SCL to JSON performed by the Middleware, as described in Section 4, were realized through the package available in [32], and encoders/decoders were implemented in Java.

XAMPP v3.2.4 was adopted for the local SCL Repository inside the Middleware [33].

The MQTT Broker was realized using Mosquitto 1.6.12 [34]. The implementation of the MQTT Publisher inside the Middleware was realized using Eclipse Paho Java Client [35].

As noted previously, in order to perform a validation of the software implementation of the IEC 61850 Web Platform, several tests on real scenarios have been carried out. The industrial partners of the PASCAL project provided access to IEC 61850 Servers that maintain information collected by real distributed energy resources (DERs). The IEC 61850 Web Platform implemented by the authors was used to realize the access to these servers, as described in Section 4. The industrial partners made a huge number of tests, requesting the access to specific data maintained by the servers, and comparing the data acquired through the platform with the real data stored in the servers. All the tests indicated the capability of the prototype to access the actual information maintained by the servers. Similar tests were conducted in the opposite direction to update data maintained by the server from the web. In this case, all the tests also indicated that the platform was able to guarantee the consistency of the updated information.

All of the conducted tests confirmed that the platform allowed interoperable access by a web application to the information maintained by IEC 61850 Servers.

## 6. Final Remarks

The paper presents a novel solution in the field of the integration of the Smart Grid and IoT technologies. This integration involves IEC 61850, RESTful Web Services, and MQTT. Section 2 presented the state-of-the-art research and the main features that make the proposal different from the existing approach in the literature.

The proposal features a RESTful web platform that is able to offer to a generic user a web service interface to IEC 61850 Servers. The web platform enables the mapping of the IEC 61850 information model into MQTT messages. Only limited knowledge is required by the Web User in order to be able to access the services offered by the platform. In particular, a very simple syntax to explore the IEC 61850 data model is requested, which does not require any knowledge about the IEC 61850 specifications or any need to use IEC 61850 communication stacks. The only technologies required for the Web User are REST and MQTT, which are considered basic technologies in the IoT. The proposal presented here was developed within a research project funded by the European Regional Development Fund. The research project features the implementation and testing of a prototype aimed to demonstrate the feasibility of the proposal. This paper provides a detailed description of the main features of the prototype software implementation and the tests carried out to validate the prototype.

Some considerations may be outlined as final remarks, as follows.

A very important question to be considered is whether the implementation of the proposed platform is worthwhile and if the advantages it provides counterbalance or exceed its (potential) complexity. The proposal presents an architecture made up of a few entities, some of which are already available; the RESTful Web Service Interface, the IEC 61850 Client inside the Middleware, and the external MQTT Broker may be realized using open-source software tools available on the web with very limited effort (as undertaken in this paper). The main effort to develop the proposed platform may be relevant to the realization of the Middleware (except for the IEC 61850 Client module). The development of the prototype realized by the authors verified that implementation issues relating to Middleware are not critical or complex. Furthermore, the availability of this platform has the advantage to allow a generic application based on REST and using MQTT and JSON protocols to access whatever information is maintained by an IEC 61850 Server. The most important thing to remember is that this access does not require any knowledge of the IEC 61850 standard nor any need to use the IEC 61850 communication stack. The authors believe that this feature is very important to enable integration of the Smart Grid with the IoT.

Another important consideration relates to the potential limits of the proposal. The authors believe that the proposal presented here may feature two limitations, pointed out as follows.

According to the proposal, the access to IEC 61850 Servers from a generic RESTful-based application must be realized through the IEC 61850 Web Platform; this dependency may be considered a limitation, although it is a very common solution when a mapping from specific standard protocols to the web must be realized. Furthermore, the advantages introduced by the proposed solution, as noted previously, are enough to counterbalance this limitation.

The other limitation of the proposal is related to the security issue. As explained in the paper, the security mechanisms implemented in the platform are user registration and user authentication. Use of the IEC 61850 Web Platform by a Web User may occur only after registration by the user with their relevant credentials (username and password). User authentication occurs after registration, and is based on the use of a signed token received by the user after the authentication request, to be used in each of the next web service synchronous requests issued to the IEC 61850 Web Platform. As specified in Section 5, the standard JSON Web Token was used for the Web User authentication [29]. The literature presents a huge number of papers dealing with the security in web service-based scenarios such as that considered in this paper; the reader may refer to [36,37,38], for example. Due to the set of secure solutions available in the current literature, and considering that security was not the main focus of this paper, the authors preferred to limit the security to the mechanisms described previously. In a real application of the proposed solution, one of the many available solutions may be adopted to improve the secure access to the platform.

## Data Availability

Not applicable.
